# 4097 Transcriptome and molecular analysis of erythropoietin-induced hypertension

**DOI:** 10.1017/cts.2020.100

**Published:** 2020-07-29

**Authors:** Nitin Kumar, Isabelle birt, Kristina L. Hunker, Santhi K. Ganesh

**Affiliations:** 1 University of Michigan School of Medicine; 2 University of Michigan

## Abstract

OBJECTIVES/GOALS: High blood-pressure (BP) is a common adverse effect of erythropoietin (EPO) therapy among patients with chronic kidney disease on hemodialysis, and even among otherwise healthy individuals who receive EPO. In human genetics, EPO is associated with not only red blood cell traits, but hypertension (HTN) as well. Currently, there is no vascular gene expression data available in the setting of EPO-induced HTN that may explain precise role of key cellular players in its hypertensive etiology. Our aim is to characterize vascular transcriptome to identify key cellular players in EPO-induced HTN. METHODS/STUDY POPULATION: 10-12 week C57BL/6 male and female mice were randomly divided into two groups 1. Vehicle (0.9% saline-VEH), 2. EPO, (N = 4). VEH and EPO were intraperitoneal administered (EPO 75U/30g, 3 times/week) for 20 days. Blood-pressure was measured non-invasively via tail-cuff plethysmography. We characterized in-vivo transcriptome response of mouse descending aorta to EPO-HTN and vehicle control group by high-throughput RNA-sequencing. RESULTS/ANTICIPATED RESULTS: Systolic blood pressure (SBP) was significantly higher in EPO treatment, compared to vehicle (males and females combined SBP VEH 116.29 ± 6.21, EPO 129.57 ± 4.59, mean ± s.d., adjusted P = 0.0012). Comparison of in-vivo transcriptional differences between vehicle and EPO-treated reveal statistically significant changes in cellular pathways consistent with hypertension such as upregulation of RAS signaling pathway and oxidative stress. In-vitro mouse aortic smooth muscle cells, EPO markedly increased phosphorylated-ERK activity, suggesting increased RAS activity. DISCUSSION/SIGNIFICANCE OF IMPACT: This study highlights the importance of previously unknown vascular key players and advances our understanding of the transcriptional events associated with EPO-induced hypertension.

